# Mechanisms of gut microbiota-immune-host interaction on glucose regulation in type 2 diabetes

**DOI:** 10.3389/fmicb.2023.1121695

**Published:** 2023-02-20

**Authors:** Yu-Dian Zhou, Feng-Xia Liang, Hao-Ran Tian, Dan Luo, Ya-Yuan Wang, Shu-Rui Yang

**Affiliations:** ^1^College of Acupuncture and Orthopedics, Hubei University of Chinese Medicine, Wuhan, Hebei, China; ^2^Department of Respiratory Wuhan No.1 Hospital, Wuhan, Hebei, China

**Keywords:** gut microbiota, intestinal immunity, blood glucose, type 2 diabetes, metabolic diseases

## Abstract

Intestinal absorption of food is one of the sources of glucose. Insulin resistance and impaired glucose tolerance caused by lifestyle and diet are the precursors of type 2 diabetes. Patients with type 2 diabetes have trouble controlling their blood sugar levels. For long-term health, strict glycemic management is necessary. Although it is thought to be well correlated with metabolic diseases like obesity, insulin resistance, and diabetes, its molecular mechanism is still not completely understood. Disturbed microbiota triggers the gut immune response to reshape the gut homeostasis. This interaction not only maintains the dynamic changes of intestinal flora, but also preserves the integrity of the intestinal barrier. Meanwhile, the microbiota establishes a systemic multiorgan dialog on the gut-brain and gut-liver axes, intestinal absorption of a high-fat diet affects the host’s feeding preference and systemic metabolism. Intervention in the gut microbiota can combat the decreased glucose tolerance and insulin sensitivity linked to metabolic diseases both centrally and peripherally. Moreover, the pharmacokinetics of oral hypoglycemic medications are also influenced by gut microbiota. The accumulation of drugs in the gut microbiota not only affects the drug efficacy, but also changes the composition and function of them, thus may help to explain individual therapeutic variances in pharmacological efficacy. Regulating gut microbiota through healthy dietary patterns or supplementing pro/prebiotics can provide guidance for lifestyle interventions in people with poor glycemic control. Traditional Chinese medicine can also be used as complementary medicine to effectively regulate intestinal homeostasis. Intestinal microbiota is becoming a new target against metabolic diseases, so more evidence is needed to elucidate the intricate microbiota-immune-host relationship, and explore the therapeutic potential of targeting intestinal microbiota.

## 1. Introduction

The earliest record of diabetes originated in ancient Egypt in 1500 BC. According to the latest diabetes map, there are about 463 million people suffering from diabetes in the world, and 116 million people in mainland China have diabetes (WHO, 2021). With the change of lifestyle, nearly 30% of people aged 30–39 have pre-diabetes (Li Y. et al., 2020). Hyperglycemia and insulin resistance (IR) can induce a variety of cardiovascular and neuropathies followed by a variety of complications. Compared with the general population, patients with diabetes reach the bowel cancer screening risk threshold 5 years earlier (Ali Khan et al., 2020), and the risk of atherosclerosis is directly doubled (Yun and Ko, 2021). The most important characteristic of diabetes is elevated blood glucose level, but intensive glucose-lowering therapy only slightly reduces vascular complications such as myocardial infarction (Newman et al., 2018; Menon et al., 2020). The academic community defines such residual risk of vascular complications after hypoglycemic treatment as “legacy effect” or “metabolic memory,” whose specific mechanism remains unclear (Chalmers and Cooper, 2008).

Most type 2 diabetes mellitus (T2D) occurs secondary to obesity and IR due to obesity (Stožer et al., 2019). Weakening of insulin secretion stimulated by glucose is a major problem in T2D (Cohrs et al., 2020). Under hyperglycemia, a large amount of electrically dependent ion channel protein (VDAC1) appears on the surface of islet β cells, causing ATP cell leakage and thus leading to β cell dysfunction (Zhang E. et al., 2019). Currently, the main methods used to combat glucose toxicity are to reduce blood glucose sources, increase insulin sensitivity, stimulate insulin secretion, and reduce kidney reabsorption of glucose (Palanisamy et al., 2018; Foretz et al., 2019; Gilbert and Pratley, 2020), which are not effective in protecting β cells or reversing diabetes status in patients diagnosed with diabetes (Aroda et al., 2017). For pre-diabetic people, lifestyle interventions and early and reasonable exercise and diet planning can effectively reduce the risk of transition to diabetes (Sampson et al., 2021). Low-fat or plant-based dietary patterns are associated with a 23% lower risk of T2D (Qian et al., 2019), and higher fruit intake, mediated by specific gut microbiota and metabolites, can also effectively reduce T2D (Jiang et al., 2020). However, the pace of life in modern society greatly limits the development of lifestyle intervention therapy, and the hypoglycemic drugs also has many side effects. Therefore, an alternative therapy that is safer, more effective and easier to adhere to is urgently needed. The human gut microbiota is composed of more than 100 trillion bacteria and more than 3 million unique genes (Hawley, 2020). The gut microbiota, by co-evolving with the host in a mutualism system, coordinates the host’s immunity, metabolism, barrier protection, and structural functions. There has been extensive research demonstrating that fecal microbiota transplantation can improve the metabolic function of obese and insulin resistant patients (Kootte et al., 2017; de Groot et al., 2020). Currently, clinical fecal microbiota transplantation methods include oral fecal microbiota transplantation capsule, nasogastric tube transplantation, endoscopic intestinal transplantation and colonoscopy transplantation (Zhang Z. et al., 2019). The metabolic benefits achieved by transplanted microbiota are strongly linked to intestinal immunity, endocrine and successful colonization of the microbiota. Therefore, this article reviews the rodent and human studies, discusses the interplay of gut microbiota and immunity, and explores the possible mechanism of gut microbiota regulating blood glucose from the perspective of gut microbiota-immune-host interaction.

## 2. Early age gut microbiota on immune function

According to statistics, there are tenfold greater intestinal microbiota than the number of cells in the human body, and the structure of individual intestinal microbiota will change in the process of interaction with host and environment (Hawley, 2020). The latest studies suggests that a small amount of *Lactobacillus* and *Staphylococcus* are present in the gut of the fetus in the second trimester of pregnancy, after isolation, it can induce and activate memory T cells in mesenteric lymph nodes *in vitro* (Ferretti et al., 2018). It is generally believed that the initial colonization of gut microbiota comes from maternal vaginal flora, faecalibacterium (Bäckhed et al., 2015) and microorganisms in breast milk (Yatsunenko et al., 2012; Ballard and Morrow, 2013). The delivery process is a critical period for establishment of the infant gut microbiota (Kennedy et al., 2021), the early establishment of gut microbiota has a direct an influence on the maturation of the intestinal immune system, as well as the metabolic function. Compared with vaginal birth, infants born by cesarean section show a lower abundance of *Bacteroides* and *Bifidobacteria* in the intestine, while a higher relative abundance of *Enterococcus faecalis*, *Haemophilus influenzae* and *Salmonella*, and an increased risk of chronic inflammatory diseases such as obesity and inflammatory bowel disease in childhood (Andersen et al., 2020). However, the development of gut microbiota composition of cesarean section infants receiving maternal fecal bacteria transplantation shows obvious similarity to that of vaginal birth infants (Korpela et al., 2020). Animal studies have also confirmed that the body weight and fat content of neonatal rats without maternal microbial environment are significantly increased, and the intestinal structure and maturity development are slower (Martinez 2nd et al., 2017), the lower abundance of *Verrucomicrobia* and *Fusobacteria* are tightly linked to the initiation of metabolic diseases such as obesity and T2D in the future (Foley et al., 2018; Liu et al., 2019). This suggests that early in life, microbes from the mother may have long-term effects on the establishment of the baby’s gut microbiota and the development of the immune system.

Accordingly, the dysregulation of immune function will also adversely affect the establishment of early gut microbiota. A large number of immune cells in breast milk can regulate the immune system of developing infants (Ballard and Morrow, 2013), among which innate lymphocytes always exist in the embryo and early life stage (Yu et al., 2018), and are the first line of immune protection for infants (Baban et al., 2018), as well as the key to intestinal microbial components and infant adaptive immunity (Artis and Spits, 2015). The immune dysfunction of preterm infants at birth can make intestinal T lymphocytes act as inflammatory mediators, leading to excessive inflammatory response when gut microbiota colonizes. In addition, T cells in the small intestinal mucosa can also regulate tissue metabolism. Mice lacking intestinal T cells have more stable microbiota structure, higher basal metabolic rate and better glucose tolerance, which can resist obesity, IR, diabetes and atherosclerosis caused by high-fat, high-sugar and high-salt diet (He et al., 2019).

## 3. The influence of intestinal immunity involving gut microbiota on blood glucose

### 3.1. Innate immunity

Intestinal innate immunity is used to resist the invasion and removal of pathogenic microorganisms of the first line of defense. It is well known that the intestinal innate immune system recognizes pathogen-associated molecular pattern (PAMP) through innate immune pattern recognition receptors (PRR), and promotes mucosal immune response by activating downstream signaling pathways and molecular events to induce the expression of anti-infective cytokines and other intestinal mucosal immune defense molecules (Kawai and Akira, 2010). Disturbance of the gut microbiota in T2D cause damage to the intestinal barrier, resulting in excessive absorption of inflammatory, which in turn aggravate systemic inflammation leading to IR (Zhao et al., 2020). Mucosal immune-mediated homeostasis of gut microbiota can effectively reduce the occurrence of intestinal barrier damage (Lin et al., 2021). Nucleotide binding oligomerization domain (Nod) receptor is a kind of PRR that exists in the cytosols of cells. Nod receptor distributed in intestinal epithelial cells can not only induce inflammation to recruit immune cells, but also induce Paneth cells to release antimicrobial molecules and directly cause epithelial cell death to remove invading pathogens (Winsor et al., 2019). Paneth cells secrete antibacterial substances and mucins that form the intestinal mucus barrier and act as the first line of immune defense against potentially harmful compounds by participating in reducing antigen exposure and bacterial influence on the immune system of the gut cells. Western-style diet that promotes obesity can cause Paneth cell defects and disturb intestinal homeostasis (Liu T. C. et al., 2021), a lack of Nod2 receptors in the gut led to more severe obesity and IR in high-fat diet-fed mice, activating Nod2 receptors prevents obesity-induced T2D by reducing the growth of *Gram-negative bacteria* in the gut (Carlos et al., 2020). Besides, the muropeptides produced in the process of gut microbiota proliferation can act on Nod2 receptors in GABA neurons in the hypothalamus through the brain-gut axis, inhibit the activity of the postsynaptic neuron, and play a role in appetite inhibition and metabolism improvement (Gabanyi et al., 2022). *In vivo*, insulin processing and secretion depend on vesicle transport (Hou et al., 2009; Zhang et al., 2020), and oligosaccharides produced by intestinal microbiota lysed by lysozyme can promote vesicle transport by binding to the immune receptor Nod2 in Paneth cells (Zhang et al., 2015). At the same time, the ligands of Nod1, which is produced in the process of intestinal microbial lysis, can enter β cells to activate Nod1 binding to vesicle surface and promote vesicle transport, thereby promoting insulin processing and transport (Zhang Q. et al., 2019).

G protein-coupled receptor (GPCR) is the largest family of membrane proteins, which can regulate human physiological activities in multiple ways, including vision, emotion, pain perception and immunity (Cohen et al., 2017). Microbiota-derived acetate promotes coordinate activation of neutrophils and ILC3 through GPR43, thereby bolsters the host’s repair responses to intestinal inflammation (Fachi et al., 2020). The absence of GPR43 in ILC3 inhibits the production of interleukin 22 (IL-22), which is required for intestinal epithelial barrier integrity and intestinal homeostasis (Chun et al., 2019). Although several studies have demonstrated that GPR43 activation improves hyperglycemia and IR in T2D mice (Huang et al., 2020; Li Y. J. et al., 2020), there was also evidence that GPR43-deficient mice fed a high-fat diet (HFD) improved glucose tolerance and impaired insulin signaling (Lu et al., 2021). At present, there is no clear consensus on the role of GPR43 in glucose metabolism, GRP43 has been shown to be a receptor for beneficial short-chain fatty acids (SCFAs) metabolized by microbes. These contradictions may be caused by downstream activation of the various G-protein subunits (Priyadarshini et al., 2015), therefore, it is of great significance to further study the role of G protein in glucose metabolism.

### 3.2. Adaptive immunity

In addition to participating in the activation of intestinal innate immunity, gut microbiota can also act on effector B and T cells through immune cell recruitment, activation and promotion of antibody secretion to combat intestinal inflammatory response and maintain intestinal metabolic homeostasis. The secretion mechanism of intestinal endocrine cells and islet endocrine cells is very similar (Habib et al., 2012). T cells are the main participants in adaptive immunity, and when they are activated, the surface insulin receptors will greatly increase (Li Y. et al., 2021). Knocking down the insulin receptors on the surface of T cells will reduce the proliferation of T cells and the secretion of inflammatory factors, and ultimately affect the adaptive immune response (Tsai et al., 2018). Dietary patterns are known to affect the gut microbial enterotypes. When human fecal bacteria who successfully lost weight through calorie restriction were transplanted into the intestines of germ-free mice, it was found that colonization of calorie-restricted bacteria could reduce white fat weight and blood glucose levels. These microbiota were positively correlated with CD4^+^ and CD8^+^ naive T cells, CD4^+^ and CD8^+^ central memory T cell subsets, and naive B cell subsets (Sbierski-Kind et al., 2022). *Akkermansia* *mucinophilus* (*AKK*) is thought to be negatively associated with the risk of obesity and T2D (Depommier et al., 2019; Xu X. et al., 2020), and *AKK* can specifically induce an acquired immune response on T cells (Ansaldo et al., 2019). Recent studies have also confirmed that *AKK* can induce dendritic cells to secrete Tumor Necrosis Factor-α (TNF-α) and IL-6 through cell membrane phospholipids, further transmitting signals to the adaptive immune system (Bae et al., 2022). The gut immune system is an essential regulator of metabolic homeostasis. The proportion and number of T helper cell 17 (Th17) in the gut of mice fed a HFD were reduced, resulting in a series of metabolic syndrome manifestations such as weight gain, IR and impaired glucose tolerance (Kawano et al., 2022). Early studies have suggested that segmented filamentous bacteria (SFB) in the gut can induce the production of Th17 cells (Ivanov et al., 2009). Th17 cells secrete IL-17 to maintain the integrity of the intestinal epithelium. Deficiency of SFB and Th17 increases the absorption of lipids by intestinal epithelial cells (Kawano et al., 2022), and epithelial absorption of dietary lipids is a known regulator of changes in blood glucose (Petersen et al., 2019). More animal experiments shows that the gene expression of the circadian clock was increased in mature type 3 natural lymphocytes (ILC3s), and the reduction of ILC3s in the intestine induced by light signals caused intestinal immune imbalance and subcutaneous fat accumulation in mice (Godinho-Silva et al., 2019). ILC3 is enriched in the gut. On the one hand, ILC3 can produce IL-17 and IL-22 to regulate the interaction with gut microbiota, and on the other hand, ILC3 can directly co-regulate adaptive immunity with T cells (Vivier et al., 2018; Sonnenberg and Hepworth, 2019).

As mentioned above, the establishment of bacteria in early life has profound effect on the development of the immune system. In neonates who take *Bifidobacterium* orally, the supernatant of fecal bacteria can directly regulate the differentiation of initial T cells (Henrick et al., 2021). Meanwhile, *Bifidobacterium* has the ability to convert thioglycosides to isothiocyanates (Bouranis et al., 2021), which has also been shown to inhibit glucose production by hepatocytes through nuclear translocation of nuclear factor-methionine 2-related factor 2 (NRF2), and to reduce fasting blood glucose and glycosylated hemoglobin in T2D patients (Zhang et al., 2022). Clinical studies have also found that, Increased abundance of *Phascolarctobacterium* and *Bacteroides stercoris* is associated with improved insulin sensitivity, and this metabolic benefit is closely related to enteroendocrine function, intestinal microecology and successful colonization of donor microbiota (Walter et al., 2018).

**Figure 1:**
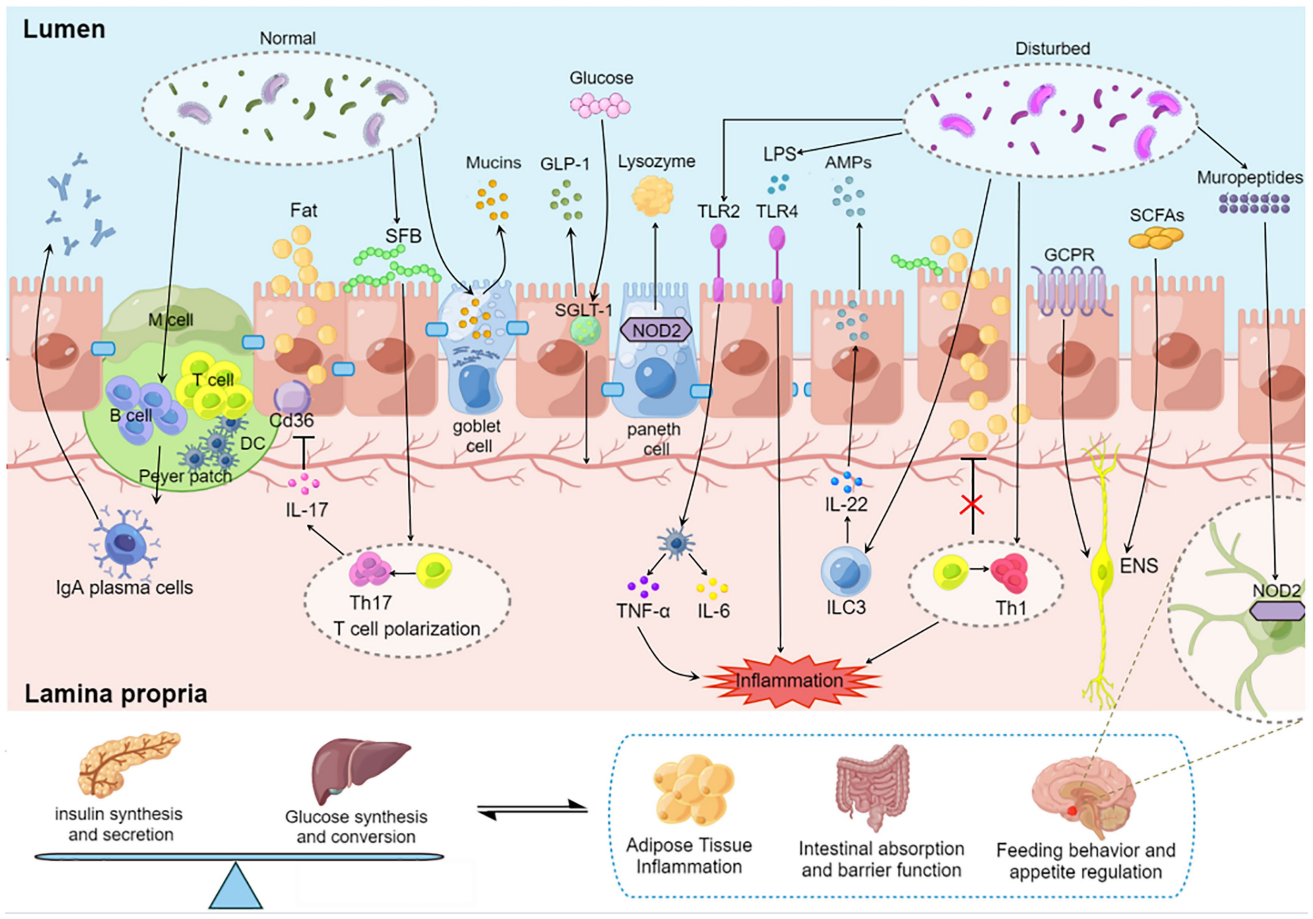
Regulation of blood glucose by gut microbiota–immunity interaction. Gut microbiota induces the maturation of gastrointestinal lymphoid tissue. Gut microbiota stimulates goblet cells to secrete mucin to protect the gut. Oligosaccharides produced by the gut microbiota bind to NOD2 receptors in Paneth cells and promote lysozyme release. SGLT-1 is influenced by the gut microbiota and regulates the balance between glucose uptake and GLP-1 release. SFB induced a Th17 response with elevated IL-17 levels, and IL-17 secreted by Th17 reduces lipid uptake and absorption by inhibiting the expression of Cd36 in epithelial cells. AKK activates TLR2 and induces dendritic cells to secrete inflammatory factors TNF-α and IL-6. Microorganisms affect the development of T cell subsets. Dysregulated microbiota release LPS to destroy the intestinal barrier, stimulate epithelial cells to release AMP through TLR4 and aggravate intestinal inflammation. SCFAs promote insulin secretion through the vagus nerve. GPCR transmits fat signals to the brain to influence feeding behavior. Mural peptides produced by gut microbes act on Nod2 receptors in GABA neurons in the hypothalamus *via* the brain-gut axis to influence appetite and metabolism. DC, dendritic cell; SFB, segmented filamentous bacteria; SGLT-1, sodium-glucose cotransporter 1; GLP-1, glucagon-like protein 1; SCFA, short chain fatty acids; GCPR, G protein-coupled receptor; ILC, native lymphocyte; TLR, Toll-like receptor; LPS, lipopolysaccharide; ENS, Enteric nervous system.

There is a complex network between insulin, gut microbiota and the immune system. Dietary fiber supplementation can maintain the stability of transplanted flora in the host. Altogether, the above studies suggest that the gut microbiota is involved in host immunomodulation. The interaction between them can regulate blood glucose from the aspects of insulin processing and transport, glucose absorption and metabolism. It can also affect the metabolism of the body from the ways of appetite and feeding behavior regulation, lipid absorption, intestinal epithelial integrity, etc (Figure 1; Table 1).

**Table 1:** Effects of gut microbiota and immune interaction on body metabolism.

Gut microbiota and its metabolites	Activation of immunity	Effects on body metabolism
Muropeptides↑ (Hou et al., 2009; Winsor et al., 2019)	Activate Nod2 receptor in GABA neurons of hypothalamus; activate the Nod1 receptor in beta cells	Increase satiety, suppress appetite; promote vesicle transport, promote insulin processing and transport
Oligosaccharides↑ (Zhang et al., 2015)	Activate Nod2 receptors in intestinal Pan’s cells	It can promote the secretion of lysozyme by Pan’s cells to dissolve pathogenic microorganisms, correct the disorder of gut microbiota, and promote the vesicle transport in islet beta cells
Lactobacillus, Staphylococcus↑	Activate mesenteric lymph node memory T cells	Enhance the bodys immune response
Lipoteichoic acid↑ (Zhang Q. et al., 2019), Akkermansia muciniphila↑ (Ansaldo et al., 2019; Depommier et al., 2019; Xu Y. et al., 2020; Sbierski-Kind et al., 2022)	Triggers mucin-2 secretion from goblet cells; activation of TLR2; and induced dendritic cells to secrete TNF-α and IL-6	Repairing the intestinal barrier; reduce intestinal inflammation; improve insulin sensitivity; lower insulinemia and total cholesterol
Proteobacteria↓, Bacteroidetes↑ (Wang et al., 2020)	Reduced the levels of intestinal effector memory CD8+ T cells, intestinal memory B cells, and hepatic effector memory CD4+ and CD8+ T cells	Alleviating intestinal immune cell senescence; losing weight; improve glucose tolerance
Bifidobacterium↑ (Bouranis et al., 2021; Henrick et al., 2021; Bae et al., 2022)	Regulates the differentiation of initial T cells	Inhibit the production of glucose by liver cells and reduce fasting blood glucose and glycosylated hemoglobin
Bifidobacterium↑ (Chaix et al., 2019)	Induction of natural lymphocyte Type 3 (ILC3)	Maintain the integrity of intestinal epithelial cells, reduce intestinal inflammation, reduce the absorption of lipids by intestinal epithelial cells
Segmented filamentous bacteria↑ (Kawano et al., 2022)	Induces the production of Th17 cells	
Phascolarctobacterium，Bacteroides stercoris↑ (Walter et al., 2018)	Induced the CD8^+^ T cells	Lose weight and improve insulin sensitivity
TMA↑ (Guo et al., 2021; Yoo et al., 2021), δ-valerobetaine↑ (Liu K. H. et al., 2021)	Affects the secretion of monoamine neurotransmitters in the gut	Regulates mitochondrial fatty acid oxidation and increases lipid storage in adipose tissue and liver
Acetic acid↑ (Andersen et al., 2022; Perry et al., 2016), butyric acid↑ (Sanna et al., 2019)	induces the secretion of GLP-1 and PYY by intestinal epithelial cells	Promote insulin secretion; Reduce appetite, increase metabolism, enhance immunity

## 4. The influence of microbiota-gut-brain axis on blood glucose

**Figure 2:**
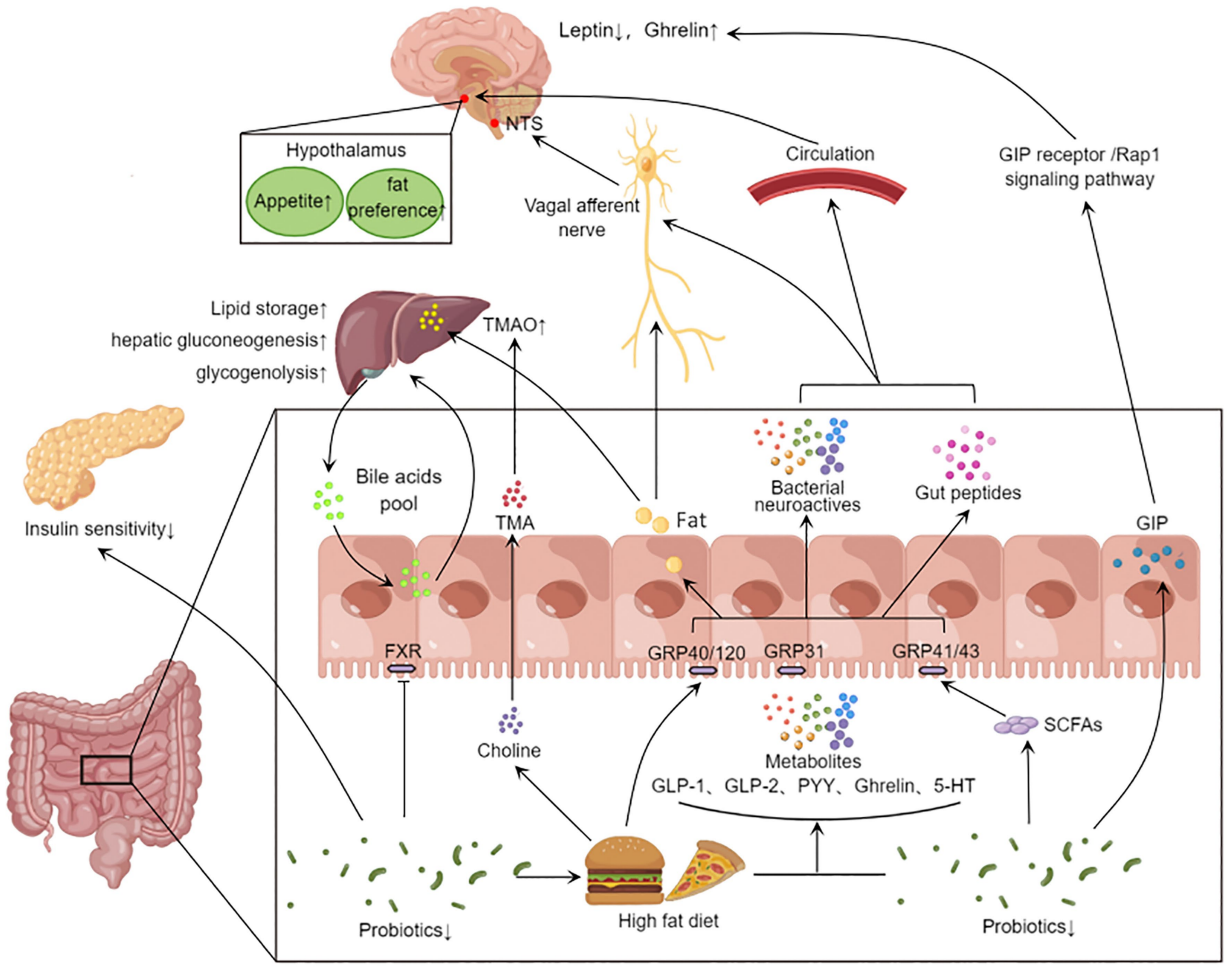
Gut microbiota affects systemic metabolism through gut-brain axis and gut-liver axis. Eating stimulates the secretion of intestinal peptides (GLP-1, GLP-2, PYY, Ghrelin, GIP, etc.) in the gut. High-fat diet reduces the abundance of beneficial bacteria, and the gut microbiota can convert food into SCFAs and 5-HT neurotransmitters. These metabolites activate different receptors and act on central appetite regulation *via* the vagus nerve or blood–brain barrier. On the other hand, the disrupted microbiota inhibits FXR signaling and affects the bile acid pool. The gut microbiota also further metabolizes fat and choline from the high-fat diet through the portal vein into the liver, affecting hepatic lipid synthesis, gluconeogenesis, and glycogenolysis. In addition, microbial metabolites can also lead to other peripheral effects. The decrease in the abundance of probiotics will reduce GLP-1 secretion, inhibit insulin signaling pathway and fat beige. GLP-1, Glucagon-like protein 1; GLP-2, glucagon-like egg 2; PYY, Peptide YY; GIP, glucose-dependent insulinotropic polypeptide; 5-HT, 5-hydroxytryptamine; SCFA, short chain fatty acids; FXR, Farnesoid X Receptor; The TMA, Trimethylamine; TMAO, Trimetlylamine oxide; NTS, nucleus tractus solitarii.

GPCR in the gut, which sense nutrients metabolized by microorganisms, are considered gastrointestinal taste receptors and important messengers in gut-brain dialog. Gut microbiota is involved in food metabolism, and neurotransmitters secreted by gut microbiota mediate the crosstalk between gut and brain (Figure 2). Similar to drug abuse, high-fat, high-sugar diets cause rapid changes in blood sugar and insulin. These peripheral signals lead to increased dopamine levels through the mesolimbic system, leading to addictive feeding behavior changes.

SCFAs can be sensed by GPR41 and GPR43, and GPR41 and GPR43 have been corroborated to regulate the expression of sodium-dependent glucose cotransporter-1 (SGLT-1) and GLUT2, thus affecting dietary glucose uptake (Ercin et al., 2022). Recent studies have found that mouse intestinal endocrine cells GPR40 and GPR120 can sense fat signals. These send signals *via* the vagus nerve to neurons in the caudal nucleus of the solitary tract of the brain, resulting in fat preference in the feeding behavior of mice (Li et al., 2022). *Parapsiella*, *Ruminococcus*, and *Trichomomonas* can affect the production of acetylcholine, and mice with impaired central cholinergic system show increased appetite and weight gain (Guo et al., 2021). The production of glucagon-like protein 1 (GLP-1), GLP-2, peotide YY (PYY), Ghrelin and 5-hydroxytryptamine (5-HT) by intestinal endocrine cells is affected by *Bacteroides*, *Bifidobacterium* and *Akkermansia* (Sandhu et al., 2017). These gastrointestinal hormones act on the hypothalamus through the blood–brain barrier and affect the brain’s judgment of appetite and feeding behavior (Martin et al., 2019). Glucose-dependent insulin stimulating peptide (GIP) secreted from the intestine can induce leptin resistance through GIP receptor/Rap1 signaling pathway (Kaneko et al., 2019).

On the one hand, gut microbes metabolize food into substances that can affect blood sugar and insulin response. On the other hand, through gastrointestinal taste receptors, enteroendocrine cells secrete a variety of regulatory peptides, such as ghrelin, GLP-1 and GIP. These regulatory peptides play a role through endocrine mechanisms and neural networks, and regulate glucose and energy metabolism by affecting metabolism, gastrointestinal motility and feeding behavior. Thus constituting the host’s vital gut-brain axis. These observations justify the need for further investigation on mechanisms that can increase intestinal taste receptor expression on enteroendocrine cells. A key goal for the future is to influence specific mediators that control insulin production by targeting changes in the microbial composition of intestinal segments, or to interfere with appetite and eating preferences for the development of therapeutics for obesity and T2D.

## 5. The influence of microbiota-gut-liver axis on blood glucose

The conversation between the gut and organs is carried out through a complex microbial-neuro-endocrine network, cocaine-and amphetamine-regulated transcript (CART^+^) neurons regulated by microorganisms can directly interact with pancreas and liver through sympathetic nerves, affecting insulin secretion and liver gluconeogenesis and glycogenolysis (Muller et al., 2020). Bile acid is an important messenger constituting enterohepatic dialog, and are synthesized in the liver and participate in the digestion of food. Most of them are reabsorbed by the ileum to the liver for re-secretion. In the intestine, microorganisms can metabolize primary bile acids into secondary bile acids and promote the activation of bile acid receptors, thereby regulating host glucose and lipid metabolism, immune response and the proliferation of intestinal pathogens (Campbell et al., 2020; Li W. et al., 2021; Figure 2). *Bifidobacterium* stimulates the secretion of intestinal GLP-1 by activating Takeda G protein-coupled receptor 5 (TGR5) and improves insulin and glucose sensitivity in mice. Bile acid chelating agent supplementation improves blood glucose control in T2D and obese patients. They also found that *Barnesiellaceae* and *Fusobacterium* can inhibit hepatic gluconeogenesis by accelerating the synthesis of cholic acid (CA) and chenodeoxycholic acid (CDCA) and activating FXR-SHP-FoxO1 pathway (Zhuang et al., 2021). Besides the gut microbiota itself, SCFAs produced by intestinal microbial fermentation can also affect blood glucose level. Some bacteria such as *Prevotella*, *Alloprevotella*, *Clostridium XlVa*, *Eubacterium* and *Intestinimonas* produce SCFAs that are beneficial to humans and could affect the secretion of GLP-1 by enteroendocrine cells and the activation of insulin signaling pathway (Zhuang et al., 2021). Of these metabolites, Butyrate can promote postprandial insulin secretion (Sanna et al., 2019). Acetate has been proven to promote insulin secretion from the islet β-cells by stimulating the vagus nerve (Perry et al., 2016)，it also reduces appetite and strengthens the immune system (Andersen et al., 2022).

The gut microbiota disrupted by HFD will affect intestinal bile acid pool and farnesol X receptor signal transduction, reduce glucose tolerance and aggravate obesity (Dantas Machado et al., 2022). It was shown that reducing the abundance of the *Coriobacteriaceae* in the gut of mice with spontaneous T2D inhibits glutamate degradation and promotes β-cell apoptosis. *Escherichia coli* metabolizes choline from a HFD into trimethylamine (TMA), which is converted to the harmful trimethylamine oxide (TMAO) in the liver *via* systemic circulation (Yoo et al., 2021). However, δ-valerobetaine, a metabolite of gut microbiota, can increase lipid storage in adipose tissue and liver by regulating mitochondrial fatty acid oxidation, leading to obesity and hepatic steatosis (Liu K. H. et al., 2021). In addition, gut microbiota and its metabolites can also direct to the liver through the portal vein and participate in liver metabolism and immunity.

This evidence explains how microbes affect glucose through the gut-liver-axis. Both the intestine and the liver are important sites for glucose and lipid absorption and metabolism, and the unique circulation between them facilitates the play of the role of gut microbiota. Compared with healthy weight, patients with T2D had more varied gut microbiota. Improving the intestinal and liver metabolic environment through intervention of specific gut microbiota may help predict and prevent T2D as soon as possible.

## 6. Feedback regulation of gut microbiota on oral hypoglycemic drugs

### 6.1. Metformin

Metformin, as the most widely used hypoglycemic drug in the world (Sanchez-Rangel and Inzucchi, 2017), was originally thought to treat diabetes mainly by inhibiting the production of glucose in the liver. Emerging evidence suggests that the biologic effects of metformin originate in the intestine. In animal experiments, oral metformin was found to have a better hypoglycemic effect than intravenous injection (Wu B. et al., 2019), and some relevant clinical studies also showed that changes in the intestinal microbiota can promote the hypoglycemic effect of metformin (de la Cuesta-Zuluaga et al., 2017). According to sequencing, oral metformin alters the gut microbiota of obese patients, however, after these microbiota were transplanted into germ-free mice, the glucose tolerance of mice was improved and the levels of SCFAs such as acetic acid and butyric acid in intestinal microbiota were increased (Rodriguez et al., 2018). The upper portion of the small intestine is an important site for glucose absorption. The SGLT-1 distributed in the upper intestine can not only absorb glucose, but also increases the release of GLP-1, which is an important molecule produced by insulin (Rhodes et al., 2022). Some researchers transplanted intestinal microbes from metformin treated HFD rats into other HFD rats, and confirmed that under the regulation of metformin, the increase of intestinal *Lactobacillus* can restore SGLT-1-mediated hypoglycemic pathway (Bauer et al., 2018). Other studies have found that imidazole propionate, as a metabolite of gut microbiota, can affect the hypoglycemic effect of metformin. It is now widely accepted that the hypoglycemic mechanism of metformin mainly lies in the activation of AMPK T172 phosphorylation (Pecinová et al., 2019), while imidazole propionate competitively inhibits AMPK T172 phosphorylation by activating AMPK S485 phosphorylation (Koh et al., 2020). Meanwhile, imidazole propionate has been shown to degrade insulin receptor substrate 1 (IRS1) by activating the mTORC1 pathway, thereby inhibiting insulin signaling (Koh et al., 2018).

It is worth noting that metformin has also been proved to be associated with fat loss, anti-cancer and anti-aging in recent years (Day et al., 2019; Miao et al., 2020). The metformin’s effect is also affected in sterile or gut microbiota disorder (Pryor et al., 2019). One interesting phenomenon is that diabetics who take metformin for a long time live longer than healthy people (Bannister et al., 2014; Mohammed et al., 2021), later studies found that taking metformin could increase the contents of *Escherichia*, *Bacteroides*, *Enterobacteriaceae* and *Citrobacter*, and agmatine produced by these bacteria is an important molecule involved in fatty acid oxidation and lipid metabolism (Pryor et al., 2019). This evidence suggests that how to accurately regulate beneficial gut microbiota may be an important direction to explore the potential efficacy of metformin.

### 6.2. Acarbose

Acarbose is an α-glucosidase inhibitor, which can competitively bind α-glucosidase in the intestine, inhibit the decomposition of starch to oligosaccharide, thereby reducing the rate of glucose synthesis and absorption, so as to achieve the effect of lowering glucose and controlling glucose. Studies have found that taking acarbose will increase the abundance of *Bifidobacterium* and *Bacteroidaceae*, resulting in changes in the intestinal environment (Baxter et al., 2019). In addition, acarbose can reduce the synthesis of deoxycholic acid and stone cholic acid by gut microbiota, and increase the synthesis of ursodeoxycholic acid. These secondary bile acids can act on the bile acid receptors in the intestine and regulate the glucose and lipid metabolism in the intestine. In contrast, strains that release enterobacteria-derived acarbose kinase in the gut and mouth, such as *Actinomyces oris*, *Solobacterium Moorei*, and *Leptotrichia Trevisanii*, can impair its hypoglycemic effect by phosphorylating acarbose. This indicates that the gut microbiota is resistant to metabolic drugs, and the long-term use of acarbose in the treatment of diabetes may be “blunted” by the gut microbiota (Balaich et al., 2021).

### 6.3. Dipeptidyl kinase-4 inhibitors

Dipeptidyl kinase-4 (DPP-4) inhibitor is a novel oral hypoglycemic agent, which can reduce the inactivation of GLP-1 *in vivo* and increase the level of endogenous GLP-1, thereby stimulating the secretion of insulin by islet β-cells and inhibiting the secretion of glucagon by islet α-cells to play a hypoglycemic effect. At present, the main DPP-4 inhibitors used in clinical practice include sitagliptin, saxagliptin, vigliptin and so on. Sitagliptin has been proved to restore the structure of gut microbiota and affect the production of SCFAs in diabetic rats (Zhang M. et al., 2019). Oral administration of sitagliptin and saxagliptin can improve the abundance of *Bacteroides* and the production of intestinal metabolite succinate in HFD mice, thereby ameliorating the decreased glucose tolerance induced by HFD (Liao et al., 2019).

With the deepening understanding of the function of gut microbiota, the influence of gut microbiota on drug metabolism has attracted increasing attention in recent years (Table 2). Notably, oral medications react with the intestinal flora before entering the bloodstream, resulting in reduced therapy activity or higher toxic metabolites (Zhang J. et al., 2019). This also explains the treatment differences of oral drugs among individuals. In the future, targeted regulation of gut microbiota and the development of microbiota agents may become a new direction of individual medical development.

**Table 2:** Effect of hypoglycemic drugs on gut microbiota.

Drugs	Gut microbiota changes	Effects on body metabolism
Metformin	Akkermansia muciniphila↑, A. muciniphila↑, Bifidobacterium↑, acetic acid and butyric acid↑ (de la Cuesta-Zuluaga et al., 2017); Lactobacillus, Akkermansia muciniphila↑ (Rodriguez et al., 2018); Lactobacillus↑ (Bauer et al., 2018); Escherichia, Bacteroides, Enterobacteriaceae and Citrobacter↑ (Pryor et al., 2019)	glucose tolerance↑, GLP-1 and PYY↑, GIP↓, LPS↓, insulin signaling↑, glucose homeostasis↑, appetite↓, weight↓, insulin sensitivity↑
Acarbose	Bifidobacterium, Bacteroidaceae↑ (Baxter et al., 2019); Actinomyces oris, Solobacterium Moorei, and Leptotrichia Trevisanii (Balaich et al., 2021)	glucose synthesis and absorption↓; deoxycholic acid and stone cholic acid↓, ursodeoxycholic acid↑;acetic acid and butyric acid↑
DPP-4 inhibitor	Bacteroides↑ (Liao et al., 2019); Proteobacteria↓ Lactobacillus↑ (Zhang M. et al., 2019)	GLP-1↑, insulin↑, Glucagon↓, succinate↑, glucose homeostasis↑

## 7. Strategies to improve blood sugar through gut microbiota

### 7.1. Dietary pattern intervention

As our understanding of the relationship between gut microbiota and metabolism deepens, people gradually began to pay attention to and design dietary patterns that can increase specific beneficial bacteria (Table 3). Dietary protein is the factor that drives the synthesis and release of secretory immunoglobulin A (sIgA), and most bacteria in the gut can induce a non-T cell dependent IgA response. High-protein diet remarkably increased the abundance of *Actinomycetes* represented by *Bifidobacterium* in the intestinal of mice, and succinate metabolized by bacteria increased sIgA secretion through extracellular vesicles, thus improving intestinal immunity (Tan et al., 2022). As a dairy product with health benefits, yogurt is generally added with *Streptococcus thermophilus* and *Lactobacillus bulgaricus*, which has been proved in many studies to be associated with the reduction of the incidence of T2D (Soedamah-Muthu and de Goede, 2018; Drouin-Chartier et al., 2019). Recently, it has been proved that branched hydroxyl acid (BCHA) in yogurt can play a hypoglycemic role by inhibiting liver glucose production and improving systemic glucose metabolic rate (Daniel et al., 2022), and the intake of yogurt and plant fermented drinks is also highly correlated with the increase of gut microbiota diversity (Wastyk et al., 2021). Dietary fiber is composed of complex carbohydrates, which is difficult for human body to digest and absorb. Instead, it is fermented by gut microbiota, causing secretion of beneficial SCFAs, and mice deficient in dietary fiber show a decrease in *Bacteroidetes* and an increase in *Proteobacteria* (Shi et al., 2021). Long-term high dietary fiber intake can significantly improve the HBA1c and gut microbiota structure in T2D patients (Zhao et al., 2018). Long-term vegetarian diets were negatively associated with T2D (Qian et al., 2019), increasing the ratio of plant foods in daily dietary pattern leads to more vitamins and minerals. Higher intake of fruit and nut affects the functions of gut microbiota in bile acid synthesis, fatty acid synthesis and metabolism (Ghosh et al., 2020; Jiang et al., 2020; Ren et al., 2020). The addition of N-3 polyunsaturated fatty acids EPA and DHA to the diet of spontaneously T2D mice increased the abundance of *Bifidobacterium*, *Lactobacillus*, *Coriobacteriaceae*, *Barnesiellaceae* and *Fusobacterium*, while *Bifidobacterium* and *Lactobacillus* has been shown to improve the endotoxemia, *Coriobacteriaceae* can accelerate degradation, thus reduce the apoptosis of β-cell (Zhuang et al., 2021). Nevertheless, the relationship between single nutrient elements and health is non-linear (Ho et al., 2020), and dietary patterns incorporating multi-nutrient components should be adopted. In general, diets high in protein, fiber, vitamins and unsaturated fatty acids are beneficial to human metabolism, the intake of ultra-processed foods, such as soft drinks, salty foods, heavy additives and processed meats, should generally be avoided (Atzeni et al., 2022).

**Table 3:** Effects of dietary patterns on gut microbiota and body metabolism.

Dietary patterns	Gut microbiota changes	Effects on body metabolism
Unsaturated fat	Bifidobacterium, Lactobacillus, Coriobacteriaceae, Barnesiellaceae, Fusobacterium↑ (Zhuang et al., 2021)	High blood glucose↓, IR↓, adipose tissue↓
Protein	Bifidobacterium↑ (Tan et al., 2022); Roseburia, Verrucomicrobia↓, Faecalibacteria, Flavonifractor↑ (Soedamah-Muthu and de Goede, 2018); Lactobacillus, streptococcus↑ (Soedamah-Muthu and de Goede, 2018; Daniel et al., 2022); Lachnospira↑ (Wastyk et al., 2021)	T2D↓, IR↓, insulin sensitivity ↑
Dietary fiber	Bacteroidetes↓, Proteobacteria↑ (Shi et al., 2021); Faecalibacterium prausnitzii, Akkermansia muciniphila↑ (Jiang et al., 2020); Ruminococcaceae, Clostridiales, Acidaminococcus↑, Fusobacterium↓ (Ghosh et al., 2020)	SCFAs↑, insulin sensitivity↑, inflammation↓, visceral fat↓, abdominal obesity↓
Vitamins and minerals	Ruminococcus↑ (Ren et al., 2020); Faecalibacterium prausnitzii, Roseburia, Eubacterium, Prevotella↑ (Ghosh et al., 2020; Ren et al., 2020)	T2D↓, obesity↓
Ultra-processed food	Alloprevotella, Negativibacillus, Prevotella↑ (Atzeni et al., 2022)	Weight↑, obesity↑, inflammation↑

Hyperglycemia frequently occurs after breakfast in some diabetic patients, this so-called “dawn phenomenon” suggests that disruptions in the circadian clock may also disrupt the circadian rhythm of insulin sensitivity. Similarly, changes in blood glucose and insulin levels caused by disturbed feeding rhythm will also affect the expression of the “circadian” related proteins (Mistlberger, 2020), and the impaired glucose tolerance and insulin sensitivity caused by disturbed feeding rhythm can be corrected by controlling eating time and changing dietary structure (Chaix et al., 2019; Crosby et al., 2019). The gut microbiota colonizes the host intestine rely on their highly dynamic nature to maintain metabolic homeostasis and shape the host’s immune system (Yang et al., 2020; Zoll et al., 2020), the feeding rhythm is one of the factors driving the changes of intestinal microbial structure and metabolism (David et al., 2014), which indicates that metabolic diseases and immune system diseases caused by circadian clock disorder are likely to be related to the changes of gut microbiota. More studies are being conducted on diet structure, dietary rhythm, fasting and calorie control. These evidences indicate that gut microbiota is highly sensitive to diet and drugs, diet and lifestyle intervention is expected to be an adjunct therapy for obesity, IR and T2D. How to target and regulate gut microbiota through diet and drug intervention needs to be further verified.

### 7.2. Fecal microbiota transplantation

Fecal microbiota transplantation (FMT) is to transplant the functional flora of the donor into the recipient to achieve disease treatment through the reconstruction of the flora. Currently, FMT has become an effective strategy for the treatment of metabolic diseases (Table 4). Fecal bacteria transplanted into T2D mice from healthy people can significantly improve the function of islet beta cells (Wang et al., 2019). At present, nasogastric tube and endoscopic intestinal transplantation are the main FMT techniques. FMT has stringent requirements for the selection of donor bacteria, and this technique also has some adverse reactions, including gastrointestinal symptoms such as abdominal distension, abdominal pain, vomiting and irregular stool (Smits et al., 2013; Drekonja et al., 2015). Furthermore, the success rate and survival time of bacterial colonization are affected by the immune response of the recipient, which may result in short curative effect time and large differences in individual benefits (de Groot et al., 2020). The gut microbiota prepared into freeze-dried capsules, combined with low-fermentation cellulose for oral administration, can improve insulin sensitivity of obese patients, prolong the colonization time of gut microbiota, and bring more long-term metabolic benefit (Mocanu et al., 2021). The interaction between flora and immunity suggests that host rejection should be considered when conducting fecal bacteria allotransplantation. When the transplanted flora is more immunocompatible with the host, the efficacy of FMT is more obvious.

**Table 4:** Effects of different interventions on gut microbiota and metabolism.

Intervention	Gut microbiota changes	Effects on body metabolism
FMT	Anaerostipes hadrus↑, Desulfovibrio ssp.↑, Alloprevotella rava↑, Coprococcus↑ (de Groot et al., 2020); hascolarcobacterium↑,Christensenellaceae↑,Bacteroidetes↑, Akkermansia muciniphila↑ (Mocanu et al., 2021)	IR↓, bile acid↑; obesity↓, insulin sensitivity↑
Pro−/ prebiotic	Lactobacillus acidophilus, Lactobacillus casei, Lactobacillus rhamnosus, Bifidobacterium bifidum, Lactococcus lactis and Streptococcus therophilus↑ (Zmora et al., 2018); Lactobacillus, Akkermansia muciniphila↑ (Wang et al., 2020); Bifidobacteria, Lactobacillus acidophilus ↑ (Sergeev et al., 2020); Lactobacillus, Bifidobacteria, Streptococcus, Enterococcus ↑ (Dai et al., 2022); fructose oligosaccharide, galactose oligosaccharide supplements↑ (Zou et al., 2018)	Intestinal barrier↑, glucose tolerance↑, IR↓, inflammation↓; Obesity↓; glucose homeostasis↑, glucose and lipid metabolism↑; feelings of satiety↑, IR↓
TCM	Ganoderma—polysaccharides (Sang et al., 2021); Cordyceps--Parabacteroides goldsteinii↑ (Wu T. R. et al., 2019); Bacteroidetes, Bifidobacteria↑ (An et al., 2019); Ginseng--Lactobacillus, Bacteroidetes↑ (Zhou et al., 2021); Gegen-qinlian--Bacteroides, Roseburia, Bifidobacteria↑ (Xu X. et al., 2020); Curcumin--Bacteroidetes, Bifidobacteria↑; Acupuncture--Bifidobacteria, Lactobacillus↑ (Yaklai et al., 2021)	IR↓, LPS↓, intestinal barrier function↑, obesity↓, pro-inflammatory cytokines↓, SCFAs↑

### 7.3. Probiotics and prebiotics

Probiotics and prebiotics are dietary supplements in which a dose of pro/prebiotics can be used to rebuild the host microbiota and have beneficial effects on host health (Table 4). Common probiotic supplements include *Lactobacillus acidophilus*, *Lactobacillus casei*, *Lactobacillus rhamnosus*, *Bifidobacterium bifidum*, *Lactococcus lactis*, and *Streptococcus therophilus* (Zmora et al., 2018). Studies have found that probiotic supplementation can trigger mucin-2 secretion by goblet cells and improve intestinal barrier damage, impaired glucose tolerance and IR caused by obesity (Wang et al., 2020). Among them, the supplementation of *Bifidobacteria* and *Lactobacillus acidophilus* for 3 months have anti-obesity effects (Sergeev et al., 2020). Several clinical randomized controlled trials have also demonstrated that the combination of multiple probiotics can reduce fasting blood glucose and improve glucose metabolism in T2D patients (Dai et al., 2022). Most of the dominant bacteria in the intestinal are anaerobic bacteria, and some facultative anaerobic bacteria such as *Escherichia coli* and *Salmonella* will multiply in aerobic conditions, thus disrupting intestinal homeostasis (Shelton et al., 2022). On the one hand, the supplement of probiotics can improve the abundance of beneficial bacteria. On the other hand, it can reduce the intestinal pH through the fermentation of carbohydrates, inhibit the proliferation of intestinal aerobic bacteria, and correct the balance of gut microbiota (Tripolt et al., 2015). Prebiotics are a kind of dietary fiber that is not easy to digest. After entering the intestine, they can be decomposed and absorbed by beneficial bacteria to promote the propagation of beneficial bacteria. Some fructose oligosaccharide and galactose oligosaccharide supplements can help create feelings of satiety, stabilize blood sugar and reduce IR (Zou et al., 2018). Inulin has also been shown to improve metabolism in mice, but there is also evidence that supplementation of refined soluble dietary fiber in the presence of gut microbiota imbalance can induce liver cancer (Singh et al., 2018). Although the mechanism has not been clarified, nutritional supplements should be carefully selected for people with disrupted gut microbiota.

### 7.4. Traditional Chinese medicine

Traditional Chinese medicine (TCM) has been treating diabetes for more than 2,000 years. Although TCM is used to combine a variety of medicinal materials into prescriptions for the treatment of diseases, modern technology has isolated many beneficial monomer components from herbs (Table 4). The effective components of Chinese herbas play a curative effect through the digestion and absorption of gastrointestinal tract, and their biological activities change with the function of gut microbiota. Polysaccharides in ganoderma and cordyceps sinensis can combat the flora disorder and intestinal barrier damage caused by obesity, and reduce the pro-inflammatory cytokines and insulin resistance index of obese mice (Wu T. R. et al., 2019; Sang et al., 2021). The non-absorbable macromolecular polysaccharides in Chinese herbs can be decomposed into small active molecules by hydrolase and reductase secreted by *Bacteroidetes* and *Bifidobacteria*, which can be better utilized by the host (An et al., 2019). Besides, Chinese herbs can affect the composition and metabolites of gut microbiota. With the increase of the dose of Gegen-qinlian decoction, the symptoms of T2D patients were improved, and the abundance of some beneficial bacteria—*Bacteroides*, *Roseburia* and *Bifidobacteria* were increased (Xu X. et al., 2020). Ginseng is rich in polysaccharides and saponins, which can enhance the reproductive ability of *Lactobacillus* and *Bacteroidetes* (Zhou et al., 2021). These extracts can also be used as prebiotics to play a role in human health. Curcumin can improve the intestinal barrier of T2D mice by increasing the abundance of *Bacteroides* and *Bifidobacterium* and reduce the entry of LPS into the blood circulation (Huang et al., 2021). In addition, acupuncture as an exogenous stimulus, the sensation of acupoints is one of the factors to judge the effect of acupuncture and moxibustion. The stimulation signal can affect the central appetite and peripheral metabolism through the neuroendocrine network (Yaklai et al., 2021). Acupuncture at some points, such as Zusanli and Zhongwan, can improve the mechanical and chemical functions of gastrointestinal tract, which may be one of the mechanisms by which acupuncture affects the flora through regulating the intestinal environment.

## 8. Discussion

The pathogenesis of T2D has not been fully verified due to its complex pathological process, which involves multi-system linkages throughout the body. The intestinal is the main place of glucose digestion and absorption. The incretin hormones secreted by intestinal epithelial cells after eating is the endogenous regulator of blood glucose. This review highlights the importance of gut microbiota and immune interaction in glucose metabolism. Gut microbiota, as a biological barrier of the intestine, plays a close biological role in the activation of intestinal immunity, and their effects on blood glucose and body metabolism have complex regulatory mechanisms (Figure 2). Future studies should focus on the activation and changes of intestinal immunity in T2D, including the specific regulatory mechanisms of gut microbiota on PRR and GPCR. Existing studies have provided evidence for the contribution of gut microbiota and immunity to glucose metabolism, but this is more correlation evidence, and more studies are needed to explore the causal link between gut microbiota and T2D.

This review also emphasizes the important role of gut microbiota and host interaction in glucose metabolism. It can be gathered that increased abundance of some beneficial bacteria—Lactobacillus, Akkermansia, Bifidobacterium, Prevotella—is associated with better insulin sensitivity and glucose tolerance, SCFAs such as butyric acid and acetic acid can enter other organs through the blood circulation and exerts positive effects on the whole-body metabolism. The multi-organ dialog, including gut-brain axis and gut-liver axis, affects appetite and feeding behavior, lipid absorption, intestinal epithelial integrity, and ultimately systematically affects glucose metabolism. According to the health state of the host, different strategies can be selected to intervene the gut microbiota. In the future, the intervention of dietary pattern and intestinal nutrition and TCM may become complementary therapies to regulate the gut microbiota of the host. Although some interventions have been used to interfere with gut microbiota, their population-wide applicability has yet to be validated in large-scale clinical trials. For T2D patients, a multi-nutrient diet that is low in fat and sugar should be part of daily life, and drug selection should be considered in combination with individual gut microbiota differences.

In the long-term evolution process, the gut microbiota, through individual adaptation and natural selection, has formed a interdependent and mutually restrictive relationship with host immunity and environment. To dig deeper in the interaction mechanism between them is helpful to understand individual differences in pharmacological intervention and provide ideas for improving drug efficacy, reducing drug side effects and developing new drugs. The use of highly stable and individual specific microbiota is conducive to the development of targeted microbiota preparations and the construction of precision medicine system.

## Author contributions

This article was primarily conceived and written by Y-DZ. H-RT helps find literature and made the tables. DL has suggested changes to this article. Y-YW explains the mechanism diagram. S-RY helped modify the format. F-XL helped revise the manuscript. The figures for this article was drawn by Figdraw. All authors contributed to the article and approved the submitted version.

## Funding

This work was supported by National Natural Science Foundation of China (Nos. 81774420 and 82274634).

## Conflict of interest

The authors declare that the research was conducted in the absence of any commercial or financial relationships that could be construed as a potential conflict of interest.

## Publisher’s note

All claims expressed in this article are solely those of the authors and do not necessarily represent those of their affiliated organizations, or those of the publisher, the editors and the reviewers. Any product that may be evaluated in this article, or claim that may be made by its manufacturer, is not guaranteed or endorsed by the publisher.
